# A comprehensive dataset on nitrate, Nitrite and dissolved organic carbon leaching losses from a 4-year Lysimeter study

**DOI:** 10.1016/j.dib.2020.106029

**Published:** 2020-07-15

**Authors:** Thierry Morvan, Charlotte Lemoine, Florian Gaillard, Gaelle Hamelin, Béatrice Trinkler, Laurence Carteaux, Patrice Petitjean, Anne Jaffrezic

**Affiliations:** a INRAE, Agrocampus Ouest, Umr Sas, 35000 Rennes, France; b Univ Rennes, CNRS, Géosciences Rennes, UMR 6118, 35000 Rennes, France

**Keywords:** Organic waste product (owp) applications, Soil tillage practices, Catch crop, Nitrate-n leaching, Dissolved organic carbon (DOC) leaching

## Abstract

This article presents a dataset on nitrate, nitrite and dissolved organic carbon (DOC) losses measured for 4 years using lysimeters at the EFELE long-term experimental site (Le Rheu, France). This ongoing long-term study was designed to provide information on effects of organic waste product (OWP) application and soil tillage on crop production, soil properties, biodiversity, greenhouse gas emissions and water quality. Forty wick-fiber lysimeters were installed at depths of 40 and 90 cm to document effects of organic and/or mineral fertilization, vegetation cover and weather conditions on dynamics of nitrate, nitrite and DOC concentrations of water collected during the drainage season (winter). These data help analyze the effects of winter plant cover (wheat vs. mustard catch crop) on these dynamics and fill a knowledge gap on effects of organic waste product supply on DOC losses. These dynamic data over several years are also of great interest for calibrating and evaluating models (e.g. STICS, APSIM, CERES).

Specification table

**Table:** 

Subject	Soil Science
Specific subject area	Effect of repeated organic waste product (OWP) applications and management practices (soil tillage practices, catch crop planting) on nitrate-N, nitrite-N and dissolved organic carbon (DOC) leaching
Type of data	Table Figure
How data were acquired	Water percolating into the soil during the drainage periods was collected by fiberglass-wick lysimeters and analyzed for nitrate, nitrite and DOC concentrations. Data from 4 consecutive drainage seasons from 2014 to 2015 to 2017–2018 are presented in this data paper.
Data format	Raw and analyzed data
Parameters for data collection	Two long-term agronomic experiments have been performed at the EFELE site since 2012: i) PROs, a randomized 4-block trial in which 5 OWP treatments are compared to a control treatment with mineral nitrogen fertilization and ii) TS/MO, a split-plot trial in which reduced vs. conventional tillage and mineral N vs. organic fertilization are studied.
Description of data collection	Nitrate, nitrite and DOC leaching were studied using lysimeters for 4 years. Six plots of the PROs trial (block 1) and 4 plots of the TS/MO trial (block 1) are equipped with fiberglass-wick lysimeters. Two pairs of lysimeters are installed in each plot at depths of 40 and 90 cm, respectively. Water was collected and sampled as a function of drainage events, with the objective of collecting water as soon as possible after drainage had stopped (within 2–7 days).
Data source location	EFELE experimental site, located in Le Rheu, France (48°06′07 N, 1°47′44 W), which is a part of the French national observatory SOERE PRO (https://www6.inra.fr/valor-pro_eng). EFELE is managed by INRAE, UMR SAS.
Data accessibility	Analyzed data are provided in this article. Raw data are deposited in a public repository. Repository name: Data INRAE Data identification number: 10.15454/RKYCLF Direct URL to data: https://doi.org/10.15454/RKYCLF

## Value of the data

• The dataset, acquired over 4 consecutive years, provides a reference for dynamics of nitrate concentration and DOC under annual crops.
• This dataset is of particular interest to soil scientists and modelers who work with soil-plant models (e.g. STICS, APSIM, CERES).
• The data help fill a knowledge gap on effects of OWP supply on DOC losses, which is little documented in the literature. Analysis of losses at 2 depths should help understand processes that influence DOC flows in the soil.
• These dynamic data over several years are particularly useful for calibrating and evaluating models.

## 1 Data description

**Fig. 1:**
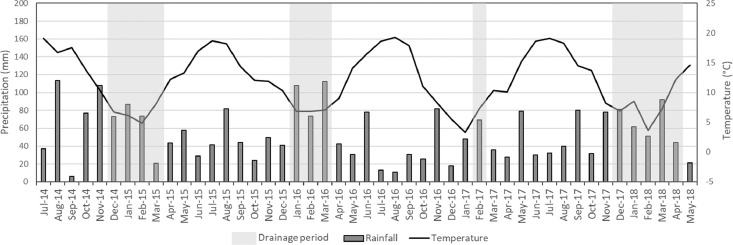
Monthly precipitation (mm) and mean temperature ( °C) from July 2014 to May 2018. gray bands indicate months during which leachate was collected.

**Fig. 2:**
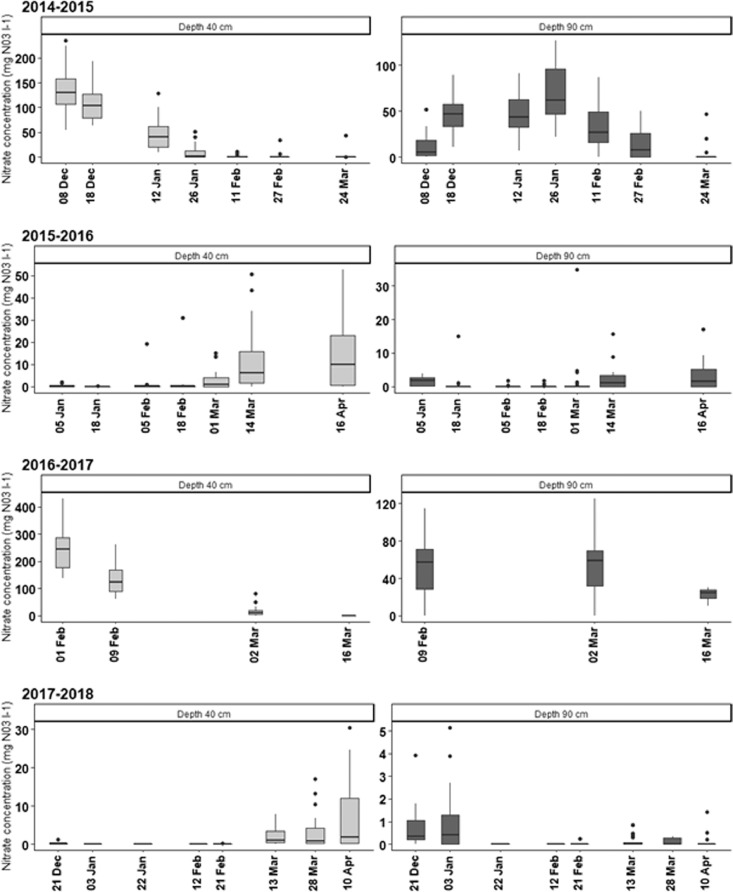
Dynamics of nitrate concentrations over the 4 drainage seasons at depths of 40 and 90 cm. Whiskers indicate 1.5 times the interquartile range.

**Fig. 3:**
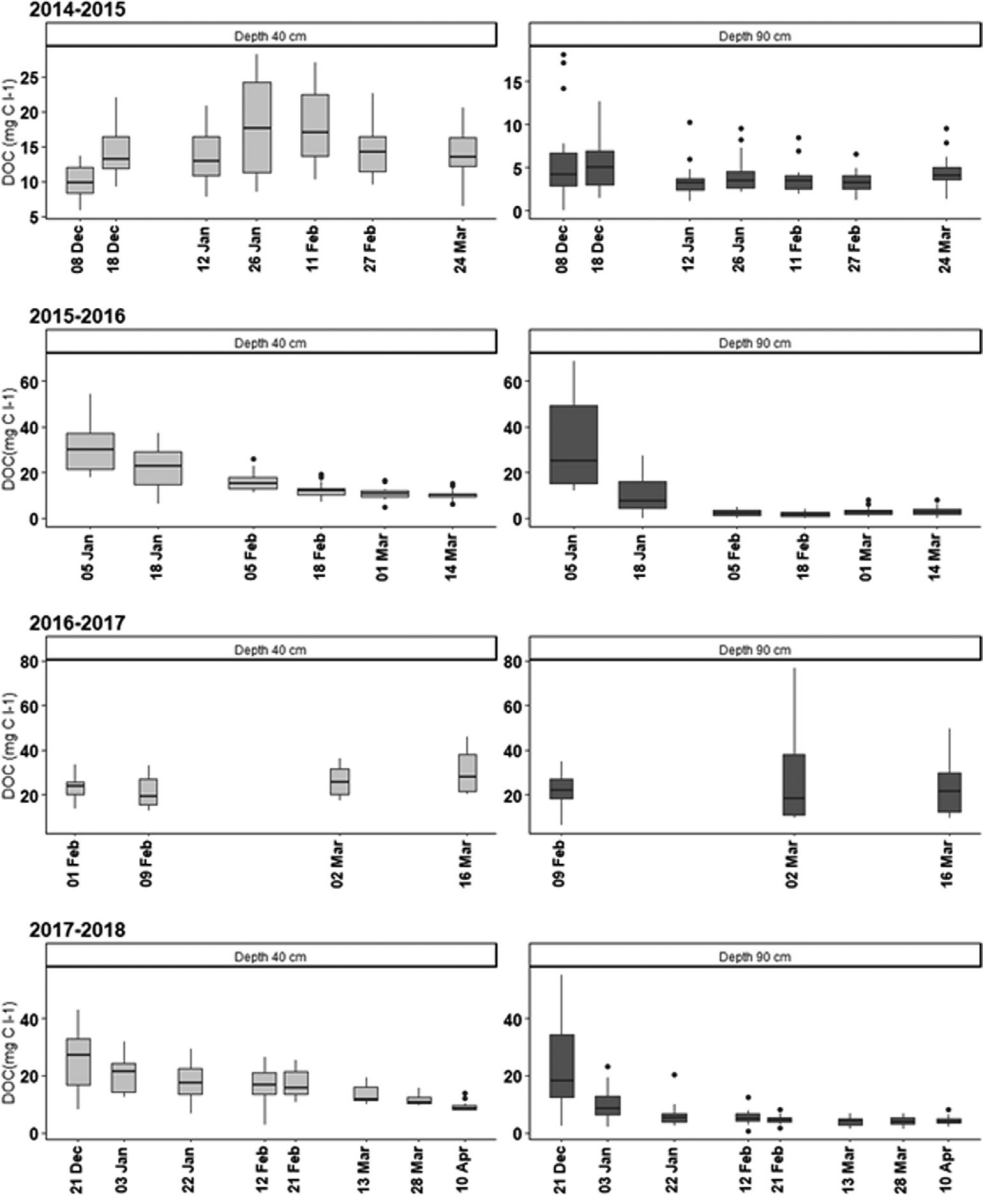
Dynamics of dissolved organic carbon (DOC) concentrations over the 4 drainage seasons at depths of 40 and 90 cm. Whiskers indicate 1.5 times the interquartile range.

This article includes descriptive tables and figures on dynamics of nitrate and dissolved organic carbon (DOC) concentrations of the water collected by EFELE's lysimeters during drainage seasons from 2014 to 2015 to 2017–2018. The data include amounts of organic waste product (OWP) and of carbon (C) and nitrogen (N) applied to the experimental treatments from 2014 to 2017 (Tables 1 and 2, respectively), dynamics of monthly rainfall and mean daily air temperature for the 4 years (Fig. 1), intra- and inter-annual dynamics of nitrate concentrations (Fig. 2) and DOC concentrations (Fig. 3), mean amounts of soil mineral N measured in early autumn and its distribution in the soil profile (Table 3).

**Table 1:** Organic waste product (OWP) application rates and corresponding rates of organic carbon (C) supplied per year from 2014 to 2017.

Parameter	Year of application	PROs trial					TS/MO trial	
		CM+*N*	PoM	CPigM+*N*	PS	PS-DIG	CT_CM	RT_CM
OWP application rates (t ha^−1^)	2014	50	3	25	25	28	50	50
	2015	0	3.4	0	32.8	27.3	0	0
	2016	50	3.4	25	23.4	40	50	50
	2017	0	3.4	0	22.5	24.4	0	0
Organic C (kg C ha^−1^)	2014	4957	327	2248	519	556	4957	4957
	2015	0	372	0	1023	694	0	0
	2016	4649	362	2220	724	361	4649	4649
	2017	0	432	0	796	641	0	0

**Table 2:** Rates of mineral and organic nitrogen (N) applied per year from 2014 to 2017 (mineral N is the sum of ammoniacal N of the organic waste product (OWP) and the N from the mineral fertilizer).

Parameter	Date	PROs trial					TS/MO trial				
		MIN	CM+*N*	PoM	CPigM+*N*	PS	PS-DIG	CT_MIN	CT_CM	RT_MIN	RT_CM
Mineral N (kg N ha^−1^)	2014	102	84	84	45	114	132	113	27.4	113	27.4
	2015	120	120	54	120	160	135	120	120	80	80
	2016	96	98	61	57	98	92	102	68	102	68
	2017	80	76	48	91	89	111	95	80	95	80
Organic N (kg N ha^−1^)	2014	0	233	23	156	30	42	0	233	0	233
	2015	0	0	21	0	47	38	0	0	0	0
	2016	0	201	27	131	40	31	0	201	0	201
	2017	0	0	29	0	34	35	0	0	0	0

**Table 3:** Mean (and 1 standard error, *n* = 39) of soil mineral nitrogen (N) (SMN) measured in early autumn from 2014 to 2017, and its distribution in the soil profile.

Horizon	6 Oct 2014	12 Oct 2015	19 Oct 2016	16 Oct 2017
0–30 cm	24.2 (0.3)	33.3 (0.2)	40.3 (0.4)	17.9 (0.1)
30–60 cm	5.8 (0.1)	20.6 (0.3)	8.9 (0.1)	18.0 (0.1)
60–90 cm	3.3 (0.07)	3.0 (0.03)	4.4 (0.06)	3.9 (0.03)

The dataset is composed of 1 PDF document and 4 Excel files that contain raw data (Table 4). It includes layouts of the PROs and TS/MO trial; daily weather data; drainage data and nitrate, nitrite and DOC concentrations measured by date and lysimeter; mineral N applications; OWP application rates and composition. The dataset is available via the Data INRAE portal.

**Table 4:** Contents of the dataset.

File name	Contents
EFELE.pdf	PROs and TS/MO trials maps - locations of the experimental treatments
Climate.xls	Precipitation (mm)
	Min. daily air temperature (°C)
	Max. daily air temperature (°C)
	Global radiation (J cm^−2^)
	Penman evapotranspiration (mm)
Lysimeter.xls	Drainage (mm)
	Cumulative drainage (mm)
	Nitrate concentration (mg NO_3_ l ^−^ ^1^)
	Nitrite concentration (mg NO_2_ l ^−^ ^1^)
	Dissolved organic carbon (mg C l ^−^ ^1^)
Mineral_N_fert.xls	Mineral N application (kg N ha^−1^)
data_OWP.xls	Application rates (t ha^−1^)
	Dry matter content (g 100 *g* ^−^ ^1^)
	Ammonium content (g N kg^−1^ raw product)
	Total N content (g N kg^−1^ raw product)
	Organic N content (g N kg^−1^ raw product)
	Organic matter content (g kg^−1^ dry matter)
	Organic C content (g C kg^−1^ dry matter)

## 2 Experimental design, materials and methods

### 2.1 Study site and experimental design

EFELE is an experimental site of the SOERE PRO network (https://www6.inra.fr/valor-pro_eng). It is used to measure long-term evolutions of agrosystems after repeated applications of organic residues derived from animal wastes that undergo a variety of treatments (e.g. none, composting, anaerobic digestion).

Two trials are underway at the EFELE experimental site (Le Rheu, France; 48°06′07 N, 1°47′44 W):

2- PROs, a complete randomized-block trial with 4 replicates, each block composed of 9 plots of 109 m^2^ each. Effects of 5 OWP on soil and water quality are compared to those of a control treatment without mineral N fertilizer (0 N) and a control treatment with mineral N fertilization (MIN). The OWP are i) cattle farmyard manure (CM), ii) pig manure composted on straw (CPigM), iii) poultry manure (PoM), iv) pig slurry (PS) and v) the digestate obtained after anaerobic digestion of the studied pig slurry (PS-DIG). Since the short-term N fertilizing value of cattle manure and composted pig manure may not be high enough to satisfy the crop's N requirements, particularly during the first few years of the trial, the OWP received a mineral N supplement (treatments CM+*N* and CPigM+*N*, respectively).
2- TS/MO, a split-plot trial with 3 replicates. Two tillage methods (conventional or reduced) are combined with mineral or organic fertilization. Four treatments are studied: conventional tillage and mineral N fertilization (CT_MIN), conventional tillage and cattle manure application (CT_CM), reduced tillage and mineral N fertilization (RT_MIN) and reduced tillage and cattle manure application (RT_MIN). Conventional tillage (CT) consists of moldboard plowing with full soil inversion to a depth of 25 cm, followed by harrowing to 12 cm before seeding. Reduced tillage (RT) consists of soil tillage with notched-edge discs, without soil inversion, to a depth of 8–10 cm.

The crop rotation consists of maize (*Zea mays* L.) and winter wheat (*Triticum aestivum* L.), with a N catch crop (CC) of white mustard (*Sinapis alba*) sown at the beginning of September, 2 months after the wheat harvest. Rates of mineral N fertilizer applied to crops were calculated using the mineral N balance-sheet method recommended in France [1] (Table 1). Poultry manure, pig slurry and the digestate of pig slurry were applied in early spring every year to the growing wheat and before the sowing of maize, while cattle manure and composted pig manure were applied every 2 years before the sowing of maize. For these 2 treatments, the N fertilization of wheat came from mineral fertilizers (Table 2).

## 3 Soil description

The soil is classified as Luvisol-Redoxisol derived from aeolian silt deposited on schist material [2]. The topsoil horizon (Ap) is 30 cm deep, and the soil is silty with a predominance of coarse silt down to a depth of 70 cm. The 70–85 cm layer defines a transition between the E and BT horizons, which is followed by a clay-accumulation horizon (BT) to a depth of ca. 120 cm.

The topsoil horizon had the following properties at the beginning of the trial: clay: 142 g 100 *g* ^−^ ^1^; silt: 708 g 100 *g* ^−^ ^1^; sand: 150 g 100 *g* ^−^ ^1^; organic C: 1.0 g 100 *g* ^−^ ^1^; organic N: 0.114 g 100 *g* ^−^ ^1^; C:N ratio: 9.6; pH: 6.1; and CaCO_3_: none.

## 4 Water sampling

In pits dug into 6 plots of block 1 of the PROs trial (treatments MIN, CM+*N*, PoM, CPigM+*N*, PS and PS-DIG) and the 4 plots of block 1 of the TS/MO trial, 2 pairs of fiberglass-wick lysimeters were installed in each plot (depths of 40 and 90 cm, respectively). Each is made of a 50 × 25 × 2 cm stainless steel plate with a fiberglass wick spread over the plate before the plate is pressed up against the soil layers above. The whole wick goes through a hole in the center of the plate, through a Tygon^Ⓡ^ tube and down to a receiving glass flask (10 l). Fourteen flasks are equipped with ultrasonic sensors (Telemecanique Sensor XX918A3C2M12) that monitor filling of the flasks, so that water can be sampled soon after drainage has stopped to minimize changes in water composition.

## 5 Climatic data

Climatic data were obtained from an automatic weather station located on the experimental site (Campbell Scientific CR1000 logger and meteorological station). Total rainfall from November to mid-April was significantly lower in 2016–2017 than that during the 3 other winters, and the drainage periods varied from 1 month in 2016–2017 to 4.5 months in 2014–2015 (Fig. 1).

## 6 Sample collection and analysis

Water was collected and sampled as a function of drainage events, with the objective of collecting water as soon as possible after drainage had stopped (within 2–7 days). Water samples were filtered at 0.45 µm and stored at 4 °C until analysis. Nitrate and nitrite concentrations were determined by ion chromatography (ICS 3000, Thermo Scientific) [3]. Samples had very low nitrite concentrations:  69% lay below the detection threshold of 0.06 mg NO_2_ l ^−^ ^1^, 6.4% lay below the quantification threshold of 0.15 mg NO_2_ l ^−^ ^1^, and only 5.4% had a concentration greater than 5 mg NO_2_ l ^−^ ^1^. Concentrations of DOC were determined with a TOC-V_CSH_ analyzer (Shimadzu Corp., Kyoto, Japan) using thermal oxidation [4].

## Ethics statement

Not applicable.

## Declaration of Competing Interest

The authors declare that they have no known competing financial interests or personal relationships which have, or could be perceived to have, influenced the work reported in this article.

## Appendix Supplementary materials

Image, application 1
